# Extending ICPC-2 PLUS terminology to develop a classification system specific for the study of chiropractic encounters

**DOI:** 10.1186/2045-709X-21-4

**Published:** 2013-01-14

**Authors:** Melanie J Charity, Simon D French, Kirsty Forsdike, Helena Britt, Barbara Polus, Jane Gunn

**Affiliations:** 1 General Practice and Primary Health Care Academic Centre, University of Melbourne, 200 Berkeley St, Carlton, VIC 3010, Australia; 2 Centre for Health Exercise and Sports Medicine, Melbourne School of Health Sciences, University of Melbourne, 200 Berkeley St, Carlton, VIC 3010, Australia; 3 Family Medicine Research Centre, Sydney School of Public Health, Sydney Medical School, The University of Sydney, Level 7, 16-18 Wentworth Street, Parramatta, NSW, 2150, Australia; 4 Research & Innovation, RMIT University, 110 Victoria Street, Melbourne, VIC, 3000, Australia

**Keywords:** Chiropractic, International classification of primary care, Classification, Clinical coding

## Abstract

**Background:**

Typically a large amount of information is collected during healthcare research and this information needs to be organised in a way that will make it manageable and to facilitate clear reporting. The Chiropractic Observation and Analysis STudy (COAST) was a cross sectional observational study that described the clinical practices of chiropractors in Victoria, Australia. To code chiropractic encounters COAST used the International Classification of Primary Care (ICPC-2) with the PLUS general practice clinical terminology to code chiropractic encounters. This paper describes the process by which a chiropractic-profession specific terminology was developed for use in research by expanding the current ICPC-2 PLUS system.

**Methods:**

The coder referred to the ICPC-2 PLUS system when coding chiropractor recorded encounter details (reasons for encounter, diagnoses/problems and processes of care). The coder used rules and conventions supplied by the Family Medicine Research Unit at the University of Sydney, the developers of the PLUS system. New chiropractic specific terms and codes were created when a relevant term was not available in ICPC-2 PLUS.

**Results:**

Information was collected from 52 chiropractors who documented 4,464 chiropractor-patient encounters. During the study, 6,225 reasons for encounter and 6,491 diagnoses/problems were documented, coded and analysed; 169 new chiropractic specific terms were added to the ICPC-2 PLUS terminology list. Most new terms were allocated to diagnoses/problems, with reasons for encounter generally well covered in the original ICPC 2 PLUS terminology: 3,074 of the 6,491 (47%) diagnoses/problems and 274 of the 6,225 (4%) reasons for encounter recorded during encounters were coded to a new term. Twenty nine new terms (17%) represented chiropractic processes of care.

**Conclusion:**

While existing ICPC-2 PLUS terminology could not fully represent chiropractic practice, adding terms specific to chiropractic enabled coding of a large number of chiropractic encounters at the desired level. Further, the new system attempted to record the diversity among chiropractic encounters while enabling generalisation for reporting where required. COAST is ongoing, and as such, any further encounters received from chiropractors will enable addition and refinement of ICPC-2 PLUS (Chiro). More research is needed into the diagnosis/problem descriptions used by chiropractors.

## Background

The chiropractic profession in Australia is an important component of the healthcare system. There are approximately 4,300 registered chiropractors in Australia [1] and each year it is estimated that over three million people, or 16% of the Australian population, consult a chiropractor at least once [2,3]. However, very little is known about chiropractic practice and there is a need to collect observational data about chiropractic clinical practice.

Classification of information in clinical practice is useful for both clinical and research purposes. In clinical situations, classification is a way of organising information and can act as a common language between health professionals [4]. In research, classification allows the information collected to be organised in a way that will make it manageable for researchers and to facilitate clear reporting. In complex cases, such as clinical healthcare research, classification involves a specialised structure of codes to link, for example, similar symptoms or similar diagnoses. In addition to organising data, classification overcomes the variance in terms used across different health disciplines and between practitioners.

The International Classification of Primary Care (ICPC) is widely used for classification in general medical practice. The first version (ICPC Version 1) [5] was published in 1987 with the second (ICPC-2) published by the World Organization of Family Doctors (WONCA) in 1997 [6]. The Family Medicine Research Centre, University of Sydney, developed an interface terminology with each term classified according to ICPC Version 1 [7]. The resultant interface terminology, ICPC-2 PLUS, is a list of terms classified to ICPC-2. ICPC-2 PLUS continues to be used, and updated, in the well-established Australian national survey of general practitioner (GP) clinical activity, the Bettering the Evaluation And Care of Health (BEACH) project, to code and classify general practice reasons for encounter, diagnoses and processes of care in Australia [8].

In addition to general medical practice, ICPC has also been used for classification in areas such as pharmacy, nutrition and traditional Chinese medicine. Van Mil et al. (1998) created a pharmacy sub-set of ICPC codes for use by community pharmacists to document complaints/diagnoses of clients when providing pharmaceutical care [9]. Van Binsbergen and Drenthen (1999) developed a system linking ICPC codes to specific nutritional information for patients. When ICPC codes were assigned to diagnoses at the time of consultation, such as heart failure with diabetes, a particular set of nutritional information was given to the patient [10]. In relation to consultations at a university traditional Chinese medicine clinic, Meier and Rogers (2006) used ICPC classification for reporting the reason for encounter and diagnosis [11].

### ICPC-2 and ICPC-2 PLUS

ICPC-2 uses three character alpha-numeric codes to classify symptoms/complaints, problems/diagnoses or processes of care. For each ICPC-2 code, the alpha component represents a chapter, or body system (such as Musculoskeletal, Cardiovascular or Neurological), and the two digit numeric component represents a concept within the body system (a symptom/complaint, problem/diagnosis or process of care). This three character code is called a rubric [6].

To allow for greater detail and specificity to be recorded in Australian general practice, the ICPC-2 PLUS terminology was developed by The Family Medicine Research Centre, University of Sydney. Each PLUS term is classified to ICPC-2. ICPC-2 PLUS uses a six character term identifier by adding a three digit number to the ICPC-2 rubric to which the term has been classified [8]. New ICPC-2 PLUS codes are created by aligning the specific problem/diagnosis, or type of care, with the most appropriate ICPC-2 rubric and then assigning the next available three digit code. As such the final three digits of the six character ICPC-2 PLUS code simply serve to identify the specific term within the rubric and do not have any other meaning. For example, the three character ICPC-2 code L01 represents ‘Neck Symptom or Complaint’. In ICPC-2 PLUS there are 11 neck-related terms in the L01 rubric that are regularly used by GPs in Australia to describe patient problems. For example ‘Pain;neck’ and ‘Stiffness;neck’ are represented in ICPC-2 PLUS as L01 004 and L01 010 respectively [8].

In research situations such as BEACH, secondary coding of collected clinical data is performed to allocate an ICPC-2 PLUS term to each reason for encounter, diagnosis/problem, and process of care. To do this, coders search an extensive keyword list with keywords linking by logic to the ICPC-2 PLUS six character codes. On selecting a keyword, the coder is presented with all available associated terms. The coder then selects the term that is considered to most closely reflect the practitioner documentation [8].

To ensure consistent coding by different researchers, the BEACH program (using ICPC-2 PLUS) has developed a set of ‘coding rules’. These rules cover situations such as coding a patient’s history of disease and coding when no reason for encounter (or diagnosis) has been documented [8]. Another rule dictates how to code when an underlying morbidity is recorded in conjunction with a secondary (associated) condition; the rule is that the underlying condition is coded rather than the secondary condition. For example, if a GP documents ‘weakness secondary to leukaemia’, the leukaemia (the primary or underlying condition) is coded rather than the weakness.

In addition to allowing coding of clinical information to a specific PLUS term, the PLUS terminology enables standardised grouping of similar concepts (or groups of concepts). This assists in the organisation of data collected for research reporting purposes. Grouping using the three digit ICPC-2 rubric provides internationally comparable data at the ICPC-2 level. However, BEACH also allows grouping using individual terms separate from any other terms within their rubric. For example, individual osteoarthritis terms from different rubrics are grouped together for reporting ‘Osteoarthritis-all’.

This coding and grouping of practice generated data enables reporting at different levels depending on the audience; reporting the body system involved (chapter), a specific condition, or a grouping of similar concepts [8]. For example, all chronic conditions can be grouped together or a specific condition (such as uncomplicated hypertension) or body system (Circulatory) can be reported.

### Aim

While the ICPC-2 classification and its associated ICPC-2 PLUS terminology are extensive, there is currently no classification system specifically designed for researching the clinical activities of the chiropractic profession. The aim of this study was to develop a research tool ICPC-2 PLUS (Chiro) by extending the current PLUS terminology with additional relevant terms for the chiropractic profession. This paper describes the process of development of a chiropractic specific terminology and classification system for use in research. This included the creation of new terms, codes and reporting groups to accurately represent chiropractic encounters.

## Methods

### Summary of Methods

The Chiropractic Observation and Analysis STudy (COAST) was a cross sectional, prospective, observational study that aimed to describe chiropractic clinical activity in Victoria, Australia. Chiropractors were trained over the telephone to complete data collection forms and recorded anonymous patient encounter data on hand written paper encounter recording forms in free text and with the use of check boxes. COAST used the ICPC-2 PLUS terminology to secondarily code free text information from chiropractor-patient encounters, but new terms were required to describe chiropractic clinical practice. In developing the chiropractic specific ICPC-2 PLUS (Chiro), researchers followed the BEACH coding rules. Upon receipt of completed COAST chiropractic clinical encounter forms, one researcher coded each form. Where necessary, and in consultation with a second researcher, new chiropractic specific terms were added to the current ICPC-2 PLUS term list. At the completion of data collection and data entry, these new terms were allocated to an ICPC-2 rubric and then grouped via these rubrics for reporting purposes. See Table 1 for the definition of a number of terms relevant to this paper.

**Table 1:** Definitions of terms used throughout this paper*

**Term**	**Definition**
**International Classification of Primary Care, Version 2 (ICPC-2):**	A classification developed by the World Organisation of Family Doctors (WONCA). ICPC chapters are based on body systems, following the principle that localisation has precedence over aetiology.
**ICPC-2 PLUS:**	An interface terminology classified to ICPC-2. ICPC-2 PLUS includes the terms used by general medical practitioners to describe three important elements of the healthcare encounter: patient reasons for encounter (RFE); problems or diagnoses managed; and process of care (such as procedures, counselling and referrals).
**Chapters (ICPC-2):**	The main divisions within ICPC-2. There are 17 chapters primarily representing the body systems.
**Code:**	In ICPC-2 this is a 3-digit alphanumeric code. In ICPC-2 PLUS this is a 6-digit alpha numeric code made up of the ICPC-2 code followed by a three digit term identifier.
**Diagnosis/problem:**	A statement of the healthcare provider’s understanding of a health problem presented by a patient, family or community. Healthcare providers are instructed to record at the most specific level possible from the information available at the time. It may be limited to the level of symptoms.
**Encounter:**	Any professional interchange between a patient and a healthcare provider.
**Keywords:**	These are used as the links to access the term in ICPC-2 PLUS. Keywords may be terms, synonyms, acronyms, etcetera, and are linked to ICPC-2 PLUS terms to facilitate data entry. Multiple keywords can be linked to a single term.
**Groups:**	A collection of ICPC-2 diagnosis/problem rubrics created for reporting purposes.
**Process of care:**	An action the health provider carries out related to the encounter, including treatment/care provided, advice given, referral to another provider, and orders for pathology or imaging.
**Reason(s) for encounter (RFEs):**	The subjective reason(s) given by the patient for seeing or contacting the healthcare provider. These can be expressed in terms of a symptom, diagnosis or the need for a service.
**Rubric:**	The title of an individual code in ICPC-2.
**Term:**	In the PLUS terminology, this is a structured expression of the free text description recorded by the clinician. There are currently approximately 8,000 terms within the ICPC-2 PLUS system. Terms may represent a disease (e.g. hypertension), a symptom (e.g. cough) or a procedure (e.g. dressing).

*Adapted from General practice activity in Australia 2011–12 [12].

### Term allocation and creation

The BEACH coding rules (using ICPC-2 PLUS) were used to guide the coding of the chiropractic terms handwritten on the encounter forms. A maximum of three reasons for encounter and three diagnoses/problems could be documented at each chiropractic-patient encounter. When more than three reasons for encounter or diagnoses/problems were provided on the encounter form, the first three recorded were used. When a reason for encounter or diagnosis/problem was repeated on an encounter form, it was only recorded once.

**Figure 1:**
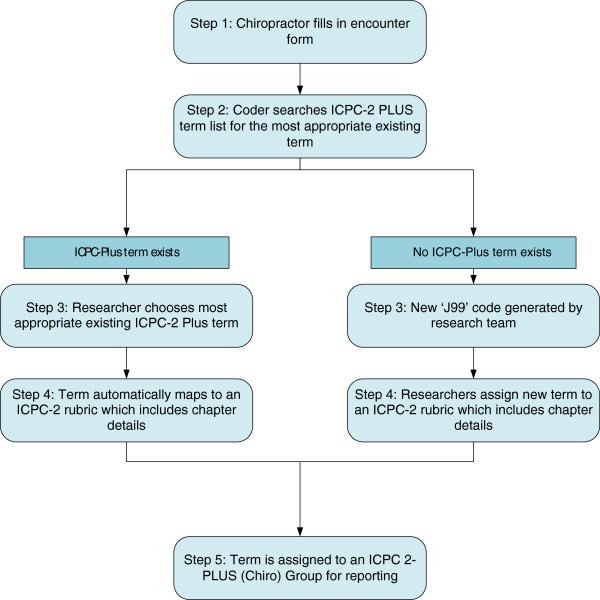
An overview of the coding procedure used in this study.

Where a reason for encounter, problem, or process of care was documented that had no corresponding ICPC-2 PLUS term, a new term (and code) was created. In line with the ICPC-2 PLUS structured format, each new code contained two parts. The first three characters of each new code were ‘J99’ to identify it as a new code not yet classified to ICPC-2 and the last three digits provided a unique numeric identifier. Using ‘J99’ ensured these new codes could not be mistaken for existing codes as there is no ‘J’ chapter in ICPC-2. See Figure 1 for an overview of the coding procedure.

Two methods were used to create new terms for reasons for encounter, diagnoses/problems and processes of care. First, a list of anticipated terms were generated by the research team. When a reason for encounter or diagnosis/problem was common in chiropractic practice, but was not represented in ICPC-2 PLUS (such as ‘chiropractic subluxation’), a ‘J-code’ was generated *before* data entry and coding. In these cases, the research team created a list of chiropractic specific diagnosis/problem terms (and related codes) that they anticipated would be recorded during the COAST encounters. These new terms represented a chiropractic specific diagnosis/problem and a site. These sites were uniform across each of the new terms, so each problem would have the same site options available. For example, terms created for problems with the wrist allowed the choice of ‘Chiropractic subluxation;wrist’, ‘Dysfunction;wrist’ or ‘Restriction/Fixation;wrist’.

Second, if an unanticipated reason for encounter, diagnosis/problem or process of care was identified during COAST data entry for which there was no existing ICPC-2 PLUS term, and no relevant term in the anticipated list, an additional term (and corresponding J-code) was created. This was done by the coder during data entry and later discussed with the research team at a coding meeting. In each case the research team discussed the information documented by the chiropractor on the encounter form, possible ICPC-2 PLUS term options, and whether a new J-code was required. Examples of terms created in this way included the reason for encounter ‘Wellbeing’ and the diagnosis/problem ‘Piriformis Syndrome’. Because these new terms were developed to describe chiropractic practice, there was no restriction on the generation of new terms for problems/diagnoses or procedures. Further, there was no attempt to merge terms that had similar meanings, e.g. joint dysfunction and manipulable lesion, as these terms come together through their classification at a later stage at rubric level.

Any new terms generated during COAST that the research team considered to be relevant to general practice were submitted to the Family Medicine Research Centre, The University of Sydney, for consideration as additions to ICPC-2 PLUS updates.

### Classifying new chiropractic terms to ICPC-2

In classifying the new chiropractic terms to ICPC-2, researchers identified the most appropriate ICPC-2 rubric for each term. ICPC-2 PLUS Keyword and Rubric Indices were searched for terms similar to the new chiropractic term. For example, the new chiropractic term ‘Dysfunction;sacroiliac joint’ was classified to the ICPC-2 rubric L03 (Low back symptom/complaint) using the PLUS term ‘Dysfunction; joint’ as a guide for chapter allocation. The term was then mapped to L03, as this rubric was more site specific than the more general L20 (Joint symptom/complaint not otherwise specified).

### Grouping

The COAST research team identified that the existing ICPC-2 classes, together with the additional PLUS reporting groups, were not suited for use in reporting common reasons for encounter and diagnoses/problems in chiropractic practice. As such, entirely new COAST-specific reporting groups were devised which were based only upon data collected during the study and not ICPC-2 grouping conventions. In addition, groups were developed only at the ICPC-2 rubric level rather than using separate PLUS terms as is sometimes done in the BEACH study.

The groups were created specifically for reporting to chiropractors, and were devised in one of two ways. The first was to group problems to a site. For example, all problems related to the shoulder were grouped to ‘Shoulder Problem’. The second grouping approach was to group all types of a problem (such as headache or depression) to one group. Each group was mutually exclusive, with no rubric being present in more than one group.

In this way all J99 codes could be grouped together with existing ICPC-2 PLUS terms for reporting through their link to an ICPC-2 rubric. For example, the J-code ‘Spinal Subluxation Syndrome’ was grouped together with ICPC-2 PLUS terms ‘Dysfunction Spine’ under our new group ‘Spinal Problem’.

## Results

**Figure 2:**
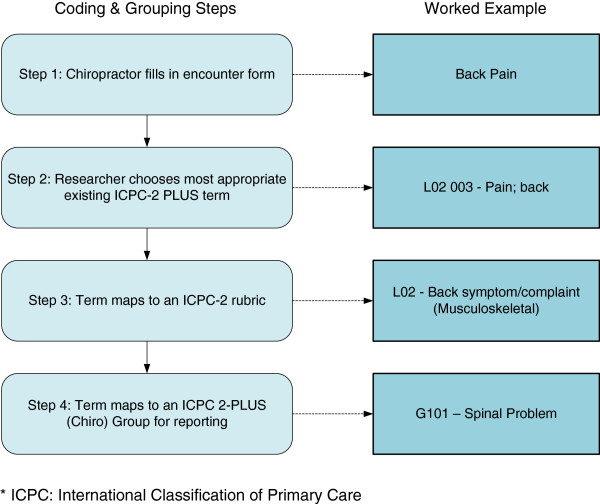
Coding and Grouping process for an example of a reason for encounter (Back Pain).

**Figure 3:**
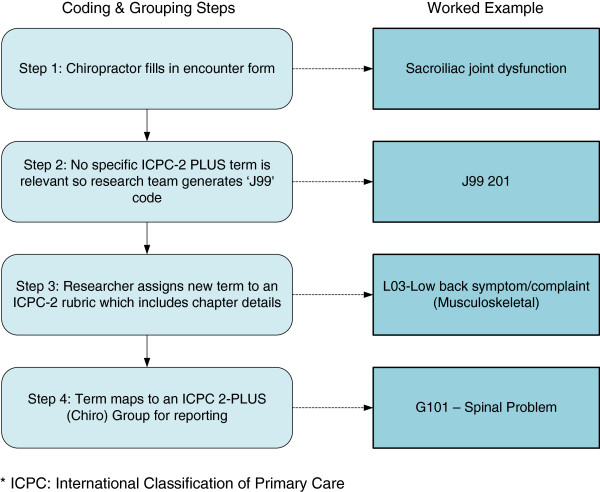
Coding and Grouping process for an example of a diagnosis/problem (Sacroiliac Joint Dysfunction).

Full results of COAST will be reported elsewhere. In brief, information was collected from 52 chiropractors (45% response rate) with 4,464 chiropractor-patient encounters documented, including 6,225 reasons for encounter and 6,491 diagnoses/problems, which were coded and analysed during the study. Figures 2 and 3 show worked examples of common encounter form entries by chiropractors and the subsequent coding and grouping process. Figure 2 shows the coding and grouping process of a reason for encounter recorded as back pain; Figure 3 shows the coding and grouping process of a diagnosis of sacroiliac joint dysfunction. Table 2 provides a full list of groups created during COAST. This table also details the ICPC-2 rubrics that were combined to form the groups.

**Table 2:** COAST Diagnosis/Problem groups and associated ICPC-2 rubrics of these groups

**Diagnosis/Problem group**	**ICPC-2 rubric**
**CG101 - OTHER BACK PROBLEM**	L02 Back symptom/complaint
	L03 Low back symptom/complaint
	L20 Joint symptom/complaint NOS
	L84 Back syndrome without radiating pain
**CG102 - MUSCLE PROBLEM**	L18 Pain, muscle
	L19 Muscle symptom/complaint NOS
	
**CG103 - BACK SYNDROME WITH RADIATING PAIN**	L86 Back syndrome with radiating pain
	N99 Neurological disease, other
**CG104 - HEALTH MAINTENANCE/ PREVENTIVE CARE**	A98 Health maintenance/preventative medicine
	A31 Check up
**CG105 - HEADACHE**	N01 Headache
	N95 Tension headache
**CG106 - NECK PROBLEM**	L01 Neck symptom/complaint
	L83 Neck syndrome
**CG107 - KYPHOSIS & SCOLIOSIS**	L85 Acquired deformity of spine
**CG108 - SHOULDER PROBLEM**	L08 Shoulder symptom/complaint
	L92 Shoulder syndrome
**CG109 - ANKLE PROBLEM**	L16 Ankle symptom/complaint
	L77 Sprains & strains of ankle
**CG110 - DEPRESSION**	P76 Depressive disorder
**CG111 - NERVE RELATED PROBLEM**	N94 Peripheral neuritis/neuropathy
	N29 Neurological symptom/complaint, other
**CG112 - OSTEOARTHROSIS, OTHER (not spine)**	L91 Osteoarthritis, other
	L89 Osteoarthritis of hip
	L90 Osteoarthritis of knee
**CG113 - VERTIGO/DIZZINESS**	N17 Vertigo/dizziness
	H82 Vertiginous syndromes
**CG114 - FEEDING PROBLEM OR IRRITABLE INFANT/CHILD**	T04 Feeding problem of infant/child
	A16 Irritable infant

In COAST, 169 new chiropractic specific terms were generated and added to the ICPC-2 PLUS term list. See the Additional file 1 for the full list of new terms. The majority of new terms were created for diagnoses/problems (140 of the 169; 83%). COAST reasons for encounter were generally well covered in the existing ICPC-2 PLUS terms with only four new terms created (2%). Other new terms represented processes of care (29 of the 169; 17%): 19 terms were generated for chiropractic methods of care and 10 terms were created for clinical advice and education given during an encounter.

The 169 new terms were used in a large proportion of the encounters recorded in COAST: 274 of the 6,225 (4%) recorded reasons for encounter, and 3,074 of the 6,491 (47%) recorded diagnoses/problems. New terms for chiropractic methods of care covered 10,855 (72%) of the total 15,179 methods of care recorded and new terms for clinical advice and education were used in coding 9% of recorded recommendations (298 of the 3,564 times a recommendation was recorded). Most (n=150, 89%) of the 169 new chiropractic terms were classified into the Musculoskeletal ICPC-2 chapter, followed by the General and Unspecified chapter (n=12, 7%).

Of the new terms generated, the most commonly used were those for techniques and care provided (e.g. ‘Manual Adjustment’ n= 4,250; and ‘Activator Instrument’ n=1,715). The most common new term for problem/diagnosis was ‘Chiropractic Subluxation’ (n=389). Fifty seven of the terms generated in anticipation of their use in chiropractic clinical practice were not recorded by the chiropractors in COAST. For a full list of new terms generated and their frequency of occurrence in the COAST data see the Additional file 1.

Fourteen reporting groups were generated using combinations of diagnostic terms via their rubrics. Of the 6,491 times a diagnosis was recorded by a chiropractor during COAST, 5,407 of these were grouped into one of the 14 COAST specific reporting groups. The remaining 1,084 remained as individual terms.

While the majority of terms created during COAST were chiropractic specific, some (such as ‘cervicogenic headache’ and ‘advice about footwear’) were considered to be relevant to general medical practice. Nine terms were submitted to the Family Medicine Research Centre, The University of Sydney, for consideration of addition to ICPC-2 PLUS. Eight of these were accepted and added to a subsequent update of ICPC-2 PLUS.

## Discussion

To accurately record chiropractic encounters, the creation of a large number of new terms was required. This study has shown that by adding chiropractic specific terms to the ICPC-2 PLUS terminology, it is possible to code a large number of chiropractic encounters to enable classification and reporting of chiropractic encounters to the desired level. However this is a work in progress and further data collection will require the addition of new terms.

Although existing ICPC-2 PLUS terms mostly covered the reason for the encounters and processes of care, the PLUS terms were not as successful in representing the diagnoses/problems recorded by chiropractors. Just under half of the total diagnoses/problems recorded in COAST were coded using newly created chiropractic specific terms.

The strength of this study came from using the well-established ICPC-2 PLUS terminology as a base and then adding to this to meet chiropractic specific needs. A large number of chiropractor specific terms were added to record chiropractic encounters. General practice and chiropractic are different in their scope so this had been expected. Using the ICPC-2 PLUS process allowed the straightforward creation of these new terms and then enabled these to be grouped together for ease of reporting.

The new terms generated in this study are a reflection of terms used by chiropractors in practice to represent what occurred in their patient encounters. Having a term assigned does not mean the diagnosis/problem can be substantiated by evidence, it simply means that one or more chiropractors used the term to record their patient encounters. More research is needed into the diagnosis/problem descriptions used by chiropractors and the level of evidence that supports the existence of the condition the chiropractors labeled. This issue has been extensively examined in the general medical practice setting, including that a definitive diagnosis is not apparent in about half of general practitioners’ consultations, that many patients present to general practice without a serious physical disorder, and that there is wide variance in the way general practitioners describe the diagnosis/problem under management [12].

This study highlighted the wide range of terms used in documentation of chiropractic encounters. This resulted in separate terms being created for what essentially could be considered the same diagnosis/problem. All new terms were mapped to ICPC-2 rubrics and chapters, so the inter-clinician variance in terms used in clinical practice is reduced when reported at these levels, where like terms are classified to the one rubric.

While a consultation process took place among the members of the research team to determine if a new term should be created, 169 new terms were still required. We assume that any further documentation of chiropractic encounters will require the generation of additional terms, and possible merging of the terms already generated, particularly the terms that were not used by the chiropractors in COAST. Future research in this area should include investigation into the terms used in chiropractic to distinguish synonyms from separate terms. A more extensive consultation process with members of the chiropractic profession would potentially allow synonyms to be identified and linked to one term rather than to have several separate terms. For example, Restriction/Fixation;pelvis may be linked to the PLUS term ‘Dysfunction;pelvis’ rather than be a separate term.

Two examples of new chiropractic terms generated in COAST highlight the different meanings of the same term used in the general medical practice profession and the chiropractic profession. First, the term ‘subluxation’ is already present in ICPC-2 PLUS in the L80 chapter ‘Dislocation/Subluxation’. However, this term is listed under the accepted medical definition of subluxation, that is, a partial dislocation. Some chiropractors use this term in a different context hence the series of terms related to ‘chiropractic subluxation’ were generated (see Additional file 1) [13]. Second, in BEACH the sacroiliac joint is not considered a moving joint, like a wrist joint, and sacroiliac recordings are classified in the rubric ‘L03: Low Back Symptom/Complaint’, because the sacroiliac joint is regarded as part of the back. However, some chiropractors consider that the sacroiliac joint is a distinct moving joint hence a series of new codes were generated in COAST to represent this (see Additional file 1) [14].

The allocation of ICPC-2 rubrics to the new chiropractic specific terms generated in COAST was done primarily for reporting purposes. Investigators had anticipated that any terms created during COAST may be difficult to assign to a chapter, as there can be disagreement as to which body system the diagnosis/problem belongs to. In most cases rubric allocation (including chapter) was straight forward, such as allocating ‘Dysfunction; joint; sacroiliac’ to the Musculoskeletal chapter using ‘Dysfunction; joint’ as a reference guide. However, in some cases rubric selection was more subjective and investigators acknowledge that other researchers may allocate different ICPC-2 rubrics to the J99 codes.

Using the COAST grouping process made it possible to report both the distribution of individual diagnoses/problems relevant to a chiropractic audience and also to the wider health community by using broader groups. The existing groups used by BEACH are general medical practitioner focused; for example Hypertension, Neoplasm and Abdominal Pain. Although the existing groups did include musculoskeletal groups such as Osteoarthritis and Sprains/Strains, in some cases the ICPC-2 PLUS group did not include terms a chiropractor would use. For example, the ICPC-2 PLUS group ‘Back Complaints (all)’ did not include the rubric L20 ‘Joint Symptom/complaint Not Otherwise Specified’ which was considered essential by the research team to include for chiropractic reporting.

Special consideration was required when assigning rubrics to COAST reporting groups, particularly as the majority of the groups were derived from newly created ‘J-codes’. Great care had been taken when classifying new chiropractic terms to ICPC-2; however, with each allocation of an ICPC-2 rubric to a COAST group, a ‘double check’ of the rubric was made. This ensured that the ‘J-code’ had been classified to the most appropriate rubric and that the rubric was assigned to the most appropriate group according to the COAST data. In this way the research team produced the groups they felt were most relevant to chiropractic. For example, to better report chiropractic care, the reporting group ‘Health Maintenance/Preventative Care’ combined any ICPC-2 PLUS term that included ‘Health Maintenance’ and ‘Check Up’ with the J99 code of ‘Wellbeing’.

It should be noted that the term ‘Problem’ has been used to name the COAST groups rather than ‘Symptom’ or ‘Complaint’. Within chiropractic clinical encounters, there is often no symptom or complaint as the reason for encounter, as shown by our large number of encounters being recorded as wellbeing and health maintenance visits. The COAST research team considered that the profession would be more accepting of the alternate title for reporting of results.

Researchers who wish to use the new ICPC-2 PLUS (Chiro) need to be aware of its limitations. The chiropractic version of ICPC-2 PLUS only contains terms recorded by the 52 participants in the COAST study who recorded 4,464 chiropractor-patient encounters, including recording details of 6,225 reasons for encounter and 6,491 diagnoses. Expansion of this study to a wider group of participants would be expected to result in additional terms added to the classification system.

Further, COAST specific reporting groups are not transferable to other studies, because they only include the ICPC-2 PLUS terms used in this study, plus the newly generated chiropractic terms. This is especially true because the COAST groups were created at the rubric level rather than at the term level. For example the COAST group ‘CG103-Back syndrome with radiating pain’ included all terms allocated to the rubric N99 ‘Neurological disease, other’. In the COAST data, only the terms ‘Neuralgia’ (N99 014) and ‘Radiculopathy’ (N99 038) were present from the whole N99 rubric. In the PLUS terminology, there are currently 34 terms allocated to the N99 rubric, including terms such as ‘Narcolepsy’ (N99 013) and ‘Encephalopathy’ (N99 042) which are not relevant in the ‘CG103-Back syndrome with radiating pain’ group.

A comprehensive chiropractic grouping tool would require each of the ICPC-2 PLUS terms to be considered for each of the COAST groups. In some cases, this would result in individual terms within a rubric assigned to different groups. For example, neuralgia might be grouped to ‘CG103-Back syndrome with radiating pain’, while Narcolepsy might not be assigned to a chiropractic group. More work is needed before this grouping can be used by other research teams.

When previous studies have used ICPC in their research, the ICPC classification was used as required for each study’s particular needs. The focus of Meier and Rogers’ (2006) study of Traditional Chinese Medicine encounters was to develop data management and reporting guidelines [11]. While that study demonstrated the use of ICPC outside a general medical practice setting, it did not add to ICPC by producing new classes specific to Traditional Chinese Medicine. Similarly, Van Mil et al. generated a pharmacy specific classification system and provided a subset of pharmacy specific ICPC codes rather than develop a terminology that was then classified to ICPC [9].

The production of ICPC-2 PLUS (Chiro) for COAST differed in two main ways from these previous studies. COAST used ICPC-2 PLUS to develop the system rather than ICPC-2; this provided coders with a large number of chiropractic relevant terms already present within the terminology. In addition, COAST was able to create new terms specific to chiropractic rather than only using those available. By using the ICPC-2 PLUS system, researchers had a wider range of keywords to search when assigning terms to reasons for encounter, diagnoses and procedures. Although using terms specifically relevant to general practice, the ICPC-2 PLUS keyword list was suited to coding information documented at chiropractic encounters. This was shown by the low percentage of new terms that were created to accurately describe reasons for encounter.

When terms relevant only to chiropractic were not present on the ICPC-2 PLUS term list, researchers were able to add new terms. This enabled a significant number of problems identified by chiropractors to be recorded that would have otherwise been placed under a non-specific term if forced to fit into the existing system. The research team had anticipated the need for new chiropractic specific terms due to the differing styles of practice and the wide range of terminology used in the profession.

## Conclusion

This is the first published chiropractic specific classification system that has been generated for reporting chiropractic clinical encounters. The research team set out to produce a system specific to chiropractic which could be used as a research tool. This is a first step in the long-term development of ICPC-2 PLUS (Chiro). COAST is ongoing, and as such, any further encounters received from chiropractors will enable addition and refinement of ICPC-2 PLUS (Chiro). We will continue to build the terminology and further develop the reporting groups as new data from a wider range of chiropractors becomes available. Development of a robust terminology and chiropractic specific classification will enable researchers to study information particular to chiropractors, using specific descriptions to accurately represent chiropractic encounters, while allowing reporting of findings to the wider health community. More research is needed into the diagnosis/problem descriptions used by chiropractors.

## Competing interests

SF is an Associate Editor with Chiropractic & Manual Therapies and had no involvement in the editorial process for this paper. Otherwise, the authors declare that they have no competing interests.

## Authors’ contributions

SF, MC and HB conceived the study. SF, JG and BP applied for funding for the study. SF, MC and KF participated in the study design and coordination. MC and SF drafted the manuscript, with significant input from HB. All authors read and approved the final manuscript.

## Supplementary Material

Additional file 1 Full list of new chiropractic specific terms generated during COAST, their frequency in the study, and the ICPC-2 rubric and chapter they were mapped to. Click here for file
