# Supplementary material for: Extending ICPC-2 PLUS terminology to develop a classification system specific for the study of chiropractic encounters
Source: Chiropr Man Therap. 2013 Jan 14;21:4. doi: 10.1186/2045-709X-21-4 (PMC3563572; doi:10.1186/2045-709X-21-4)
Supplement: Additional file 1 — Full list of new chiropractic specific terms generated during COAST, their frequency in the study, and the ICPC-2 rubric and chapter they were mapped to. [file 2045-709X-21-4-S1.pdf]

	J99 assigned	Suggested ICPC description	Frequency*	ICPC-2 rubric	ICPC-2 chapter
1	J99 057	Manual adjustment (technique)	4250	L57	Musculoskeletal
2	J99 059	Activator Instrument (technique)	1715	L57	Musculoskeletal
3	J99 062	Drop Piece (technique)	1412	L57	Musculoskeletal
4	J99 060	Blocks (technique)	1265	L57	Musculoskeletal
5	J99 058	Mobilisation (technique)	922	L57	Musculoskeletal
6	J99 001	Chiropractic subluxation	839	L02	Musculoskeletal
7	J99 102	Chiropractic subluxation;spine	389	L02	Musculoskeletal
8	J99 003	Wellness care	337	A98	General & unspecified
9	J99 103	Chiropractic subluxation; spine;cervical	294	L01	Musculoskeletal
10	J99 007	Pain; back; mechanical	239	L02	Musculoskeletal
11	J99 061	Flexion Distraction (technique)	221	L57	Musculoskeletal
12	J99 201	Dysfunction;sacroiliac joint	208	L03	Musculoskeletal
13	J99 104	Chiropractic subluxation; spine;thoracic	195	L02	Musculoskeletal
14	J99 105	Chiropractic subluxation; spine;lumbar	148	L03	Musculoskeletal
15	J99 005	Headache; cervicogenic	135	N01	Neurological
16	J99 033	Cranial (technique)	115	L57	Musculoskeletal
17	J99 030	Impulse IQ (technique)	96	L57	Musculoskeletal
18	J99 008	Pain; neck; mechanical	90	L01	Musculoskeletal
19	J99 034	Increase water	87	A45	General & unspecified
20	J99 106	Chiropractic subluxation;pelvis	68	L03	Musculoskeletal
21	J99 025	Structural assymetry	65	D18	Digestive
22	J99 002	Acupuncture; electro (technique)	57	A59	General & unspecified
23	J99 202	Dysfunction;rib joint	55	L02	Musculoskeletal
24	J99 016	Myofascial Release (technique)	49	L14	Musculoskeletal
25	J99 013	Neuro Emotional Complex (NEC)	42	L84	Musculoskeletal
26	J99 041	PNF (technique)	42	L57	Musculoskeletal
27	J99 212	Dysfunction;cranial	41	L29	Musculoskeletal
28	J99 017	Crossed syndrome(upper/lower)	36	L57	Musculoskeletal
29	J99 063	therapy; laser	33	L57	Musculoskeletal
30	J99 045	No treatment (technique)	30	A69	General & unspecified
31	J99 042	Contract relax (technique)	26	L57	Musculoskeletal
32	J99 107	Chiropractic subluxation;sacroiliac joint	26	L03	Musculoskeletal
33	J99 210	Dysfunction;foot	26	L17	Musculoskeletal
34	J99 031	Charette extremity technique	24	L57	Musculoskeletal
35	J99 209	Dysfunction;ankle	23	L16	Musculoskeletal
36	J99 108	Chiropractic subluxation;rib joint	20	L02	Musculoskeletal
37	J99 118	Chiropractic subluxation; spine;sacrum	19	L03	Musculoskeletal
38	J99 027	Integration brain (hemisphere, frontal)	18	L29	Musculoskeletal
39	J99 032	Toggle(technique)	18	L57	Musculoskeletal
40	J99 044	Traction (technique)	18	L57	Musculoskeletal
41	J99 054	cranio-sacral therapy (technique)	18	L57	Musculoskeletal
42	J99 211	Dysfunction;TMJ	16	L07	Musculoskeletal
43	J99 039	Symptom/complaint Immunity	15	B29	Blood, Blood forming organs & Immune Mechanism
44	J99 015	Piriformis syndrome	14	N89	Neurological
45	J99 014	Migraine; cervicogenic	13	A29	General & unspecified
46	J99 006	Impingement; nerve/Pressure; nerve	12	N94	Neurological
47	J99 019	Instability;pelvic	12	L29	Musculoskeletal
48	J99 056	advice/education: homeopathy (technique)	12	A45	General & unspecified
49	J99 036	Advice/edu footwear	11	A45	General & unspecified
50	J99 043	Lymphatic drainage (technique)	11	L57	Musculoskeletal
51	J99 253	Restriction/Fixation; spine;cervical	11	L01	Musculoskeletal
52	J99 029	Leopold's technique	9	L29	Musculoskeletal
53	J99 038	Short leg	9	L14	Musculoskeletal
54	J99 052	Visceral (technique)	9	L57	Musculoskeletal
55	J99 200	Dysfunction;pelvis	9	L03	Musculoskeletal
56	J99 101	Chiropractic subluxation;facet joint	8	L02	Musculoskeletal
57	J99 208	Dysfunction;knee	8	L15	Musculoskeletal
58	J99 050	Trigger points (as Dx)	7	L19	Musculoskeletal
59	J99 109	Chiropractic subluxation;shoulder	7	L08	Musculoskeletal
60	J99 203	Dysfunction;shoulder	7	L08	Musculoskeletal
61	J99 035	Avoid self manipulation	6	A45	General & unspecified

62	J99 113	Chiropractic subluxation;hip	6	L13	Musculoskeletal
63	J99 207	Dysfunction;hip	6	L13	Musculoskeletal
64	J99 254	Restriction/Fixation; spine;thoracic	6	L02	Musculoskeletal
65	J99 116	Chiropractic subluxation;foot	5	L17	Musculoskeletal
66	J99 306	Misalignment/malalignment;pelvis	5	L03	Musculoskeletal
67	J99 037	Advice/education; sleep	4	A45	General & unspecified
68	J99 048	Therapy;interferential	4	L57	Musculoskeletal
69	J99 051	Iliotibial band(ITB) syndrome	4	L29	Musculoskeletal
70	J99 053	Delayed development;neural	4	N29	Neurological
71	J99 111	Chiropractic subluxation;wrist	4	L11	Musculoskeletal
72	J99 115	Chiropractic subluxation;ankle	4	L16	Musculoskeletal
73	J99 119	Chiropractic subluxation;leg	4	L14	Musculoskeletal
74	J99 121	Chiropractic subluxation;cranial	4	L29	Musculoskeletal
75	J99 500	Irritation;facet (joint);cervical	4	L01	Musculoskeletal
76	J99 022	Pain; foot; mechanical	3	L15	Musculoskeletal
77	J99 040	Chemo(therapy) support	3	A79	General & unspecified
78	J99 205	Dysfunction;wrist	3	L11	Musculoskeletal
79	J99 255	Restriction/Fixation; spine;lumbar	3	L03	Musculoskeletal
80	J99 004	Problem; recurring	2	A29	General & unspecified
81	J99 023	Pain; SI; mechanical	2	L17	Musculoskeletal
82	J99 024	Ileocaecal Valve Syndrome	2	L03	Musculoskeletal
83	J99 047	Congestion;lymphatic	2	B29	Blood, Blood forming organs & Immune Mechanism
84	J99 055	therapy; G5 vibration (technique)	2	L57	Musculoskeletal
85	J99 110	Chiropractic subluxation;elbow	2	L10	Musculoskeletal
86	J99 120	Chiropractic subluxation;arm	2	L09	Musculoskeletal
87	J99 213	Dysfunction;leg	2	L14	Musculoskeletal
88	J99 250	Restriction/Fixation;joint	2	L20	Musculoskeletal
89	J99 252	Restriction/Fixation;spine	2	L02	Musculoskeletal
90	J99 263	Restriction/Fixation;hip	2	L13	Musculoskeletal
91	J99 266	Restriction/Fixation;foot	2	L17	Musculoskeletal
92	J99 010	Pain; hip; mechanical	1	L02	Musculoskeletal
93	J99 020	Pain; shoulder; mechanical	1	L03	Musculoskeletal
94	J99 021	Pain; knee; mechanical	1	L08	Musculoskeletal
95	J99 026	SOT category	1	L29	Musculoskeletal
96	J99 028	Unstable spine	1	N29	Neurological
97	J99 046	Muscle energy technique	1	L57	Musculoskeletal
98	J99 049	Deconditioning Syndrome	1	L29	Musculoskeletal
99	J99 114	Chiropractic subluxation;knee	1	L15	Musculoskeletal
100	J99 117	Chiropractic subluxation;TMJ	1	L07	Musculoskeletal
101	J99 204	Dysfunction;elbow	1	L10	Musculoskeletal
102	J99 206	Dysfunction;hand	1	L12	Musculoskeletal
103	J99 251	Restriction/Fixation;facet joint	1	L02	Musculoskeletal
104	J99 257	Restriction/Fixation;sacroiliac joint	1	L03	Musculoskeletal
105	J99 258	Restriction/Fixation;rib joint	1	L02	Musculoskeletal
106	J99 261	Restriction/Fixation;wrist	1	L11	Musculoskeletal
107	J99 302	Misalignment/malalignment;spine	1	L02	Musculoskeletal
108	J99 305	Misalignment/malalignment; spine;lumbar	1	L03	Musculoskeletal
109	J99 355	Distortion; spine;lumbar	1	L03	Musculoskeletal
110	J99 356	Distortion;pelvis	1	L03	Musculoskeletal
111	J99 503	Irritation;costovertebral	1	L02	Musculoskeletal
112	J99 012	Degeneration; Sacroiliac	0	L13	Musculoskeletal
113	J99 100	Chiropractic subluxation;joint	0	L20	Musculoskeletal
114	J99 112	Chiropractic subluxation;hand	0	L12	Musculoskeletal
115	J99 256	Restriction/Fixation;pelvis	0	L03	Musculoskeletal
116	J99 259	Restriction/Fixation;shoulder	0	L08	Musculoskeletal
117	J99 260	Restriction/Fixation;elbow	0	L10	Musculoskeletal
118	J99 262	Restriction/Fixation;hand	0	L12	Musculoskeletal
119	J99 264	Restriction/Fixation;knee	0	L15	Musculoskeletal
120	J99 265	Restriction/Fixation;ankle	0	L16	Musculoskeletal
121	J99 300	Misalignment/malalignment;joint	0	L20	Musculoskeletal
122	J99 301	Misalignment/malalignment;facet joint	0	L02	Musculoskeletal
123	J99 303	Misalignment/malalignment; spine;cervical	0	L01	Musculoskeletal

124	J99 304	Misalignment/malalignment; spine;thoracic	0	L02	Musculoskeletal
125	J99 307	Misalignment/malalignment;sacroiliac joint	0	L03	Musculoskeletal
126	J99 308	Misalignment/malalignment;rib joint	0	L02	Musculoskeletal
127	J99 309	Misalignment/malalignment;shoulder	0	L08	Musculoskeletal
128	J99 310	Misalignment/malalignment;elbow	0	L10	Musculoskeletal
129	J99 311	Misalignment/malalignment;wrist	0	L11	Musculoskeletal
130	J99 312	Misalignment/malalignment;hand	0	L12	Musculoskeletal
131	J99 313	Misalignment/malalignment;hip	0	L13	Musculoskeletal
132	J99 314	Misalignment/malalignment;knee	0	L15	Musculoskeletal
133	J99 315	Misalignment/malalignment;ankle	0	L16	Musculoskeletal
134	J99 316	Misalignment/malalignment;foot	0	L17	Musculoskeletal
135	J99 317	Misalignment/malalignment;coccyx	0	L03	Musculoskeletal
136	J99 350	Distortion;joint	0	L20	Musculoskeletal
137	J99 351	Distortion;facet joint	0	L02	Musculoskeletal
138	J99 352	Distortion;spine	0	L02	Musculoskeletal
139	J99 353	Distortion; spine;cervical	0	L01	Musculoskeletal
140	J99 354	Distortion; spine;thoracic	0	L02	Musculoskeletal
141	J99 357	Distortion;sacroiliac joint	0	L03	Musculoskeletal
142	J99 358	Distortion;rib joint	0	L02	Musculoskeletal
143	J99 359	Distortion;shoulder	0	L08	Musculoskeletal
144	J99 360	Distortion;elbow	0	L10	Musculoskeletal
145	J99 361	Distortion;wrist	0	L11	Musculoskeletal
146	J99 362	Distortion;hand	0	L12	Musculoskeletal
147	J99 363	Distortion;hip	0	L13	Musculoskeletal
148	J99 364	Distortion;knee	0	L15	Musculoskeletal
149	J99 365	Distortion;ankle	0	L16	Musculoskeletal
150	J99 366	Distortion;foot	0	L17	Musculoskeletal
151	J99 400	Manipulable lesion;joint	0	L20	Musculoskeletal
152	J99 401	Manipulable lesion;facet joint	0	L02	Musculoskeletal
153	J99 402	Manipulable lesion;spine	0	L02	Musculoskeletal
154	J99 403	Manipulable lesion; spine;cervical	0	L01	Musculoskeletal
155	J99 404	Manipulable lesion; spine;thoracic	0	L02	Musculoskeletal
156	J99 405	Manipulable lesion; spine;lumbar	0	L03	Musculoskeletal
157	J99 406	Manipulable lesion;pelvis	0	L03	Musculoskeletal
158	J99 407	Manipulable lesion;sacroiliac joint	0	L03	Musculoskeletal
159	J99 408	Manipulable lesion;rib joint	0	L02	Musculoskeletal
160	J99 409	Manipulable lesion;shoulder	0	L08	Musculoskeletal
161	J99 410	Manipulable lesion;elbow	0	L10	Musculoskeletal
162	J99 411	Manipulable lesion;wrist	0	L11	Musculoskeletal
163	J99 412	Manipulable lesion;hand	0	L12	Musculoskeletal
164	J99 413	Manipulable lesion;hip	0	L13	Musculoskeletal
165	J99 414	Manipulable lesion;knee	0	L15	Musculoskeletal
166	J99 415	Manipulable lesion;ankle	0	L16	Musculoskeletal
167	J99 416	Manipulable lesion;foot	0	L17	Musculoskeletal
168	J99 501	Irritation;facet (joint);thoracic	0	L02	Musculoskeletal
169	J99 502	Irritation;facet (joint);lumbar	0	L03	Musculoskeletal

* Terms with a frequency of '0' were predefined expected terms but were not used by participating chiropractors
